# Hospital and Other News

**Published:** 1922-07

**Authors:** 


### Hospital News.

In connection with St. Paul's Hospital, Holborn, a research laboratory has been established, through the generosity of Mr. Pickett, at the new hospital, now nearly completed, in Endell Street.

The well at Northampton General Hospital not only saves the institution about £40 in rates, but the corporation, at the present dry season, 30,000 gallons.

A testimonial is being given by the Committee and staff of Queen Mary's Hospital, Stratford, E., to Mr. J. Wright on his retirement after 16 years' service on the Committee.

The Downs Sanatorium, opened by the Metropolitan Asylums Board in 1913, is to be vacated and the patients moved to the new King George V. Sanatorium at Godalming, which, it is hoped, His Majesty will open.

The Country Home, Brockley Hill, Stanmore, acquired a year ago for the sun-treatment of patients in the Royal National Orthopædic Hospital, is nearly furnished, and the removal thereto of tuberculous patients is about to begin.

Dr. Eric Pritchard has been appointed Medical Director of the Infants' Hospital, Vincent Square, Westminster. The medical staff of the hospital is to be reorganised and the work widely developed, with the intention of making the hospital a centre for research and teaching in connection with infant welfare.

"Old Hastings House," formerly known as the Mansion, Hastings, has been closed after seven years. From 1915 to 1919 it was used as a Red Cross hospital, and since then by the Ministry of Pensions. The house is famous as the residence of Coventry Patmore, the poet, from 1875 to 1891. It may now become a Home of Rest for a medical mission.

"In consideration of extraordinary services," Mr. William Bonnington and Mr. Frank Taylor have been elected Honorary Life Governors of Derbyshire Royal Infirmary. Mr. Bonnington, for forty years an active worker, has done wonders for the Hospital Saturday movement. Mr. Taylor has had much success in organising local vegetable shows for the hospital, and was a pioneer in their planning.

The Fort Pitt Military Hospital, Chatham, originally part of the Thames fortifications during the Napoleonic Wars, and once the headquarters of the R.A.M.C. in the late war, is to be closed. The patients are to be moved to the Royal Naval Hospital, Chatham. The duel described in "Pickwick" was arranged in a field adjoining the hospital, and close by Dickens spent his boyhood.

The Duke of Sutherland has opened the Southfield Sanatorium at Liberton, which has been started by the Royal Victoria Hospital Tuberculosis Trust, Edinburgh. Sir Ralph Anstruther is the chairman of the committee. The hospital was opened in 1894; a farm colony was formed in 1910; and further investigations will be made at the Sanatorium Colony, where special cases, otherwise badly provided for, will be treated.

The new clinical unit in obstetrics and gynæcology at the Royal Free Hospital has completed its first year of work, and has been approved by the representatives of London University who lately inspected it. There are 45 obstetrical beds, 25 gynæcological beds, which have treated 966 and 324 cases respectively. Ten students are attached to each side of the unit, which comprises a number of clinics and a large maternity district.

Lord Tenterden, presiding at the Annual Court of the Battersea General (Anti-Vivisection) Hospital, referred sympathetically to the death of the late secretary, Mr. G. W. F. Robbins, and spoke of the keenness which the new secretary, Mr. H. W. Woolven, had brought to the task. The report referred to the new department for the Handcock treatment of children for adenoids without operation, and recorded the need of money to equip the new Out-patients' Department.

### Other News.

Sir William Treloar has been unanimously elected hon. member of the British Orthopædic Association in recognition of his work for crippled children.

The campaign on behalf of the Montreal Hospitals has, says the "Daily Telegraph's" correspondent, all but raised the $750,000 asked for.

A Professional and Business Women's Hospital League has been formed to provide free expert medical and surgical treatment in the private wards of the London, or, by special arrangements, at the provincial hospitals. The subscription to the League is 5s. per annum. Viscountess St. Cyres is the President, and Viscountess Cowdray, the Public Trustee, Miss Philippa Fawcett and Mr. Harold Marigold are the trustees. The Secretary is Miss Rawson, to whom inquiries may be addressed at 197 Edgware Road, W. 2.

For some years the Order of St. John of Jerusalem in England has maintained an ophthalmic hospital in Jerusalem. Last year there were 1,312 in-patients and 12,100 new out-patients, with 62,237 consultations and 3,001 operations. There is no lessening of contagious eye disease in Palestine, nor can this be expected until the villages and homes are properly planned and constructed, and the duty of personal cleanliness is appreciated. The number of cases attending to be supplied with spectacles showed a rapid increase, the cases numbering 760 in 1921, compared with 473 in 1920 and 269 in 1919. The Order of St. John spent £3,646 in the hospital last year, being £1,893 more than the income received.

As a result of a series of meetings and conferences of industrialists, educators and eye-experts from various parts of the United States, held under the auspices of the Eyesight Conservation Council of America, plans have been formulated to encourage eyesight conservation, particularly in industry. A campaign will be conducted for this purpose under the general direction of Mr. L. W. Wallace, Executive Secretary of the American Engineering Council of the Federated American Engineering Societies. According to "Industrial and Labour Information," issued weekly by the International Labour Office (League of Nations), it has been found that the eye is involved in 11 per cent. of all permanently disabling industrial accidents in the United States, and that approximately 25 million workers in the United States have defective vision that requires correction. Among other activities an educational campaign will be instituted to overcome the objections of workmen to the wearing of goggles, which are necessary in many employments.

Livingstone College, Leyton, which held its Commemoration Day on June 9, received last year for elementary medical training 51 students of eight nationalities, representing 26 missionary societies. The chairman, Mr. Robert Munro, M.P., Secretary of State for Scotland, said that the training given by the College was not only proper but essential for men and women who would be situated at distant outposts of the Empire, far from medical advice. He appealed to missionary societies to send their students if only for a short course. The Rev. H. F. Burroughs, of the Moravian Missionary Society, who has been working on the borders of Thibet, at a station some 16 days from a medical man, told of several instances when he had been able to do good, thanks to Livingstone College. Called to attend a boy who had fallen on a rock, he found extensive fractures of the skull, the brain being exposed, and was able to treat the lad so that he is a normal boy to-day. As to the value of the training in helping him to look after his own family, he believed that it was only through this that his little girl's life was saved. His was the only family which had come home intact from that part of the work. The Livingstone College man is an asset to the medical missionary; "a man who knows how to put on a pair of artery forceps is of far more use to him than a man with D.D. and other things after his name, whom he would probably find useless." In a small way a Livingstone College man is also an asset to the Government, which, in an epidemic of smallpox, is glad to send a supply of vaccine to a man who can use it properly. The College needs £500 to meet its present liabilities, as well as the bursaries for students to which we referred last month.

